# Physiotherapists’ use of airway clearance techniques during an acute exacerbation of bronchiectasis: a survey study

**DOI:** 10.1186/s40945-020-00097-5

**Published:** 2021-02-01

**Authors:** Jennifer Phillips, Annemarie Lee, Rodney Pope, Wayne Hing

**Affiliations:** 1 Physiotherapy, Faculty of Health Sciences and Medicine, Bond University, Gold Coast, Queensland Australia; 2 The Wesley Hospital, Uniting Care Health, Brisbane, Queensland Australia; 3 Department of Physiotherapy, School of Primary and Allied Health Care, Faculty of Medicine, Nursing and Health Sciences, Monash University, Frankston, Victoria Australia; 4 Institute for Breathing and Sleep, Melbourne, Victoria Australia; 5 Cabrini Allied Health Research and Education, Malvern, Victoria Australia; 6 School of Community Health, Charles Sturt University, Albury, New South Wales Australia

## Abstract

**Background:**

Airway clearance techniques are recommended for individuals with bronchiectasis both in stable state and during an acute exacerbation, however the current use of airway clearance techniques in the management of individuals during an acute exacerbation is unclear. The aims of this study were to establish what current physiotherapy clinical practice comprises for adults and paediatrics during an acute exacerbation of bronchiectasis; identify physiotherapist’s perceptions of the effectiveness of airway clearance techniques and identify what factors influence their treatment decisions in this population.

**Methods:**

An anonymous online survey was distributed to the members of the Australian Physiotherapy Association and Physiotherapy New Zealand between August 2016 and April 2017.

**Results:**

The survey was accessed by 130 physiotherapists and 121 of those deemed themselves eligible and consented to participate. Most participants (89%) reported prescribing airway clearance techniques for 81–100% of individuals during an acute exacerbation of bronchiectasis. The most commonly used airway clearance techniques with adults were huffing (92%), exercise (89%) and the active cycle of breathing technique (89%). The techniques perceived most effective for adults were physical exercise (100%), oscillating positive expiratory pressure devices (97%), directed huffing (95%) the active cycle of breathing technique (90%) and positive expiratory pressure (90%). The most commonly used airway clearance techniques for paediatric patients were: newborn-3 years - percussion (85%) and modified postural drainage (85%); 4–10 years - huffing (100%) and exercise (85%); 11–18 years - huffing (92%) and exercise (77%), active cycle of breathing technique (77%) and positive expiratory pressure therapy (77%). The techniques perceived most effective for paediatric patients were directed huffing (100%), percussion (100%) and positive expiratory pressure via a mask or mouthpiece (93%). The most commonly reported factors influencing choice of technique were patient clinical presentation (72%) and the presence/absence of contra-indications (72%).

**Conclusion:**

This survey demonstrates that airway clearance techniques are routinely used as part of physiotherapy management of individuals experiencing an acute exacerbation of bronchiectasis, and that choice of technique and perceived effectiveness varies depending on the age of the patient.

## Introduction

Bronchiectasis is a chronic lung condition characterised by repeated respiratory infections, productive cough, shortness of breath and decreased exercise tolerance [1, 2]. Management involves medication and physiotherapy, aiming to improve airway clearance and exercise tolerance, minimise exacerbations and improve health-related quality of life (HRQOL) [3–5]. Physiotherapy involvement in the management of bronchiectasis is supported by international guidelines which state that individuals with a chronic productive cough or difficulty clearing sputum should be taught an airway clearance technique (ACT) by a trained respiratory physiotherapist [4, 6–8]. During an acute exacerbation of bronchiectasis, intensive ACTs, including altered prescription and follow-up, are required [4].

Airway clearance techniques consist of a variety of breathing patterns with or without a device, designed to aid sputum clearance, improve ventilation and reduce cough frequency [9, 10]. Techniques include the active cycle of breathing technique (ACBT), autogenic drainage, postural drainage (PD), manual techniques, positive expiratory pressure (PEP) and oscillating PEP devices [5, 11]. Although guidelines recommend the use of ACTs, it is unclear which ACT is most effective during an acute exacerbation of bronchiectasis, and how often and for how long each technique should be implemented [12, 13].

To guide clinically relevant future research investigating the clinical efficacy of ACTs that are commonly used, it is important to know the current management of individuals with bronchiectasis. Three survey studies have investigated physiotherapists’ use of ACTs in bronchiectasis [11, 14, 15]. However, they did not focus on management of individuals with an acute exacerbation, and new guidelines for the management of individuals with bronchiectasis have subsequently been published in Australia, Brazil, Britain, and Europe [4, 6–8].

The aims of this study were to: (1) establish what current physiotherapy clinical practice in Australia and New Zealand comprises for adults and paediatrics during an acute exacerbation of bronchiectasis; (2) identify physiotherapist’s perceptions of the effectiveness of ACTs and (3) identify what factors influence their treatment decisions in this population.

## Methods

### Study design

A cross-sectional, online, survey was carried out between August 2016 and April 2017 using the Qualtrics platform (Qualtrics, Provo Utah, USA). Ethical approval was received for this research, and informed consent provided by all participants.

### Participants

Therapists were eligible to participate in the study if they were a physiotherapist who currently and routinely (in the last 12 months) treated adult or paediatric individuals during an acute exacerbation of bronchiectasis as inpatients or outpatients. The following definition of an acute exacerbation was provided to participants at the start of the survey: An acute exacerbation of bronchiectasis involves a sudden change in symptoms such as increased cough, change in sputum (volume, colour, viscosity), breathlessness, haemoptysis and/or constitutional upset (malaise, tiredness) [5].

Physiotherapists were recruited through newsletters of the professional bodies (the Australian Physiotherapy Association and Physiotherapy New Zealand), which included a link to the online survey. The electronic link took the physiotherapist to an information and consent page, and if the physiotherapist deemed themselves eligible and consented to participate, they clicked a button to proceed to the survey. The survey invitation was disseminated on four occasions, to maximise responses. Participant responses were anonymous.

### Survey design

A team of four physiotherapists developed the survey following a literature review, using a combination of new questions and questions from previous surveys investigating physiotherapy management of respiratory conditions [11, 15, 16]. The research team involved two experienced clinicians in respiratory physiotherapy, and two researchers with previous work in quantitative and qualitative research. The survey was tested for content and construct validity throughout the development process by the research team, and prior to distribution was piloted in an independent group of six experienced respiratory physiotherapists. No changes were made to the survey following the pilot, prior to distribution.

All participants were asked three questions collecting their personal demographic information. They were subsequently directed to complete additional relevant sections based on the management of adults, paediatrics, or both. Questions regarding use of ACTs in the paediatric population were split into age groups (newborn-3 years, 4–10 years and 11–18 years) due to expected differences in clinical management based on age [17]. The survey was divided into domains: (1) workplace demographics, (2) ACTs used and perceived effectiveness (3) participants’ rationales for treatment decisions regarding use of ACTs.

The workplace demographic domain (domain 1) contained six multiple choice questions and participants were able to select one response. Information regarding inpatient and outpatient settings was collected in relation to ACT prescription frequency only, and no other comparison of clinical practice in each setting was collected. Participants were asked a multiple-choice question to determine what percentage of patients with an exacerbation of bronchiectasis to whom they prescribed ACTs, with the options including “81-100%, 61-80%, 41-60%, 21-40% or 0-20%”. The domain for ACTs used (domain 2) contained three questions. These questions gathered information regarding the use of different types of ACTs and were predominantly designed as four- or five-point Likert scales (e.g. routinely, occasionally, rarely or never) to allow for comparison to previous studies. Content (such as ACTs listed) was based on previous surveys, clinical practice guidelines and research, [11, 15, 16], with an open, ‘other’, option to capture information from participants who used ACTs that were not listed. Multiple choice questions were also used to gather information regarding prescription of ACTs. The questions relating to rationales for treatment decisions (domain 3) used a combination of Likert scales, multiple choice questions with single or multiple responses allowed, and open-ended questions. Perceived effectiveness of each airway clearance technique was investigated using a Likert scale from very effective, effective, neutral, ineffective or very ineffective following the question “how effective at clearing sputum do you believe the following ACTs are for patients with an acute exacerbation of bronchiectasis”. In total, the survey included 29 questions regarding the management of adults, and 30 questions regarding paediatric management. The final version of the survey can be obtained from the authors upon request.

### Statistical analysis

Data were analysed descriptively using SPSS V.25 (SPSS, Chicago, Illinois, USA) and presented using tables and narrative text. Results were presented as frequencies and percentages of participants who responded to that question.

## Results

### Survey response

The survey was distributed widely to approximately 26,000 email addresses (Australian Physiotherapy Association membership = 23,153, Physiotherapy New Zealand membership = 3596) [18, 19]. The survey was accessed by 130 individuals between August 2016 and April 2017, and 121 of those identified themselves as eligible physiotherapists and consented to participate. Two people accessing the survey declined to consent and seven had not treated an adult or child with an acute exacerbation of bronchiectasis in the last 12 months, and were therefore ineligible and excluded. Sixty-two participants (51%) partly completed the survey, and their completed responses were included in the analysis along with responses from participants who completed the survey in its entirety. As the number of responses per question was variable, the tables included in the manuscript each state the total number of participants responding to each question.

### Participant demographics

Eligible participants were from both Australia (*n* = 106; 88%) and New Zealand (*n* = 12; 10%) and three participants accessed the survey from other countries (Table 1). All states in Australia and both islands in New Zealand were represented. Participants ranged in experience in respiratory physiotherapy (Table 2). Most participants (68%) managed only adults with an acute exacerbation of bronchiectasis, and so completed the adult section of the survey; 15% had managed only children and so completed the paediatric section of the survey, and 11% completed both sections.

**Table 1:** Participant locations (n (%); *N* = 121)

Location	Participants n (%)
Australia	
Australian Capital Territory	1 (1)
New South Wales	22 (18)
Northern Territory	2 (2)
Queensland	59 (49)
South Australia	4 (3)
Tasmania	4 (3)
Victoria	7 (6)
Western Australia	7 (6)
New Zealand	
North Island	11 (9)
South Island	1 (1)
Other	3 (2)

**Table 2:** Participant years of practice (n (%); *N*= 120)

	0–5 years	6–10 years	11–15 years	16–20 years	21 years or more
Years as a physiotherapist	33 (28%)	21 (17%)	20 (16%)	15 (13%)	31 (26%)
Years practicing in respiratory physiotherapy	48 (40%)	24 (20%)	22 (18%)	5 (4%)	21 (18%)

### Airway clearance prescription

Most participants (86%) working with adults and all participants (100%) working in paediatrics reported they prescribed ACTs in 81 to 100% of instances in which they contributed to management of an acute exacerbation of bronchiectasis. In inpatient settings, adult inpatients were most commonly treated once or twice per day (once per day 52%, twice per day 47%, three times per day 1%). In paediatric settings, inpatients were most commonly treated twice per day (once per day 5%, twice per day 86%, three times per day 9%). In outpatient settings, both adults and paediatric patients were most commonly treated once per fortnight (45 and 65% respectively), followed by once per week (28 and 20% respectively). Time spent on ACT per therapy session in an adult population was most commonly 11–15 min (34%), followed by 16–20 min (31%) and 21 or more min (27%). Time spent on ACT in each session was longer in a paediatric population, most commonly 16–20 min (58%), followed by 21 min or more (36%) then 11–15 min (6%).

### Airway clearance techniques used

The most commonly used ACTs with adults were directed huffing (92%), physical exercise (89%) and the ACBT (89%) (Table 3). Positioning (84%) and oscillating PEP devices (75%) were also used regularly. Within paediatric populations, the most commonly used ACT varied depending on age group. In the newborn-three years age group, the most commonly reported ACTs were modified PD (no head down tilt) (85%), percussion (84%) and positioning (72%) (Table 3). In the 4–10 years age group, participants reported most commonly using directed huffing (100%), physical exercise (85%) and PEP mask or mouthpiece (75%) (Table 3). In the 11–18 years age group participants reported most commonly using directed huffing (93%), the ACBT (77%), PEP mouthpiece or mask (77%) and physical exercise (77%) (Table 3).

**Table 3:** Frequency of use of ACTs (n (%); N physiotherapists treating adults = 64; N physiotherapists treating paediatrics = 13)

Airway clearance technique	Very often/always or often	Sometimes	Rarely/never
Active Cycle of Breathing Technique			
Newborn – 3 yrs	1 (8)	1 (8)	11 (84)
4–10 years	8 (67)	2 (17)	2 (16)
11–18 years	10 (77)	1 (8)	2 (16)
Adult	57 (89)	3 (5)	4 (6)
Autogenic Drainage			
Newborn – 3 yrs	2 (16)	2 (17)	8 (67)
4–10 years	1 (8)	5 (38)	7 (54)
11–18 years	5 (39)	5 (39)	3 (23)
Adult	20 (31)	17 (27)	27 (42)
Deep breathing exercises			
Newborn – 3 yrs	4 (39)	2 (15)	6 (46)
4–10 years	9 (70)	1 (8)	3 (23)
11–18 years	7 (54)	3 (23)	3 (23)
Adult	41 (65)	12 (19)	10 (16)
Directed huffing (Forced Expiration Technique)			
Newborn – 3 yrs	5 (46)	2 (16)	5 (39)
4–10 years	13 (100)	0 (0)	0 (0)
11–18 years	12 (93)	0 (0)	1 (7)
Adult	59 (92)	5 (8)	0 (0)
Postural Drainage (Head Down Tilt)			
Newborn – 3 yrs	1 (8)	1 (8)	11 (85)
4–10 years	1 (8)	1 (8)	11 (85)
11–18 years	0 (0)	2 (15)	11 (85)
Adult	5 (8)	12 (19)	46 (73)
Modified Postural Drainage (no Head Down Tilt)			
Newborn – 3 yrs	11 (85)	1 (8)	1 (8)
4–10 years	6 (46)	4 (31)	3 (23)
11–18 years	3 (23)	2 (15)	8 (62)
Adult	30 (47)	17 (26)	17 (26)
Manual vibration			
Newborn – 3 yrs	7 (54)	4 (31)	2 (16)
4–10 years	8 (62)	3 (23)	2 (16)
11–18 years	4 (31)	3 (23)	6 (46)
Adult	30 (47)	18 (28)	16 (25)
Mechanical vibration (Vest)			
Newborn – 3 yrs	0 (0)	0 (0)	12 (100)
4–10 years	0 (0)	0 (0)	13 (100)
11–18 years	0 (0)	0 (0)	13 (100)
Adult	5 (8)	0 (0)	58 (92)
Oscillating Positive Expiratory Pressure device			
Newborn – 3 yrs	0 (0)	0 (0)	12 (100)
4–10 years	6 (46)	2 (23)	4 (31)
11–18 years	7 (54)	1 (8)	5 (39)
Adult	48 (75)	12 (19)	4 (6)
Oscillating Positive Expiratory Pressure bubble/bottle			
Newborn – 3 yrs	6 (47)	1 (8)	6 (47)
4–10 years	9 (69)	1 (8)	3 (23)
11–18 years	7 (54)	1 (8)	5 (39)
Adult	41 (65)	8 (13)	14 (22)
Positive Expiratory Pressure mask or mouthpiece			
Newborn – 3 yrs	4 (32)	5 (37)	4 (31)
4–10 years	9 (75)	1 (8)	2 (16)
11–18 years	10 (77)	1 (8)	2 (16)
Adult	26 (41)	21 (33)	16 (26)
Percussion			
Newborn – 3 yrs	11 (84)	1 (8)	1 (8)
4–10 years	6 (47)	4 (31)	3 (23)
11–18 years	3 (23)	4 (31)	6 (46)
Adult	24 (38)	21 (33)	18 (29)
Physical exercise			
Newborn – 3 yrs	8 (62)	0 (0)	5 (38)
4–10 years	11 (85)	2 (16)	0 (0)
11–18 years	10 (77)	2 (15)	1 (8)
Adult	57 (89)	5 (8)	2 (3)
Positioning			
Newborn – 3 yrs	10 (72)	1 (8)	3 (22)
4–10 years	6 (46)	5 (38)	2 (16)
11–18 years	5 (38)	6 (46)	2 (15)
Adult	54 (84)	5 (8)	5 (8)
Sustained maximal inspiration			
Newborn – 3 yrs	NA	NA	NA
4–10 years	NA	NA	NA
11–18 years	NA	NA	NA
Adult	19 (30)	12 (19)	33 (51)

*NA* Not asked

### Perceived effectiveness of ACTs

Participants working with adults perceived the most effective techniques were physical exercise (100%), oscillating PEP devices (97%), directed huffing (95%), the ACBT (90%) and PEP mask or mouthpiece (90%) (Table 4). All participants believed that physical exercise, PEP therapy, directed huffing and oscillating PEP devices were effective (Table 4). The ACTs considered least effective in adults were PD including head down tilt (23%), sustained maximal inspiration (18%) and mechanical vibration (18%).

**Table 4:** Perceived effectiveness of ACTs (n (%); N physiotherapists treating adults = 64, N physiotherapists treating paediatrics = 13)

Technique	Very effective or effective	Neutral or N/A	Ineffective or not effective at all
Active cycle of breathing technique			
Adult	58 (90)	5 (8)	1 (2)
Paediatric	12 (92)	1 (8)	0 (0)
Autogenic Drainage			
Adult	49 (76)	14 (22)	1 (2)
Paediatric	10 (78)	2 (15)	1 (8)
Deep breathing exercises			
Adult	34 (53)	24 (38)	6 (9)
Paediatric	8 (61)	5 (38)	0 (0)
Directed huffing (Forced Expiration Technique)			
Adult	61 (95)	3 (5)	0 (0)
Paediatric	12 (100)	0 (0)	0 (0)
Postural Drainage (Head Down Tilt)			
Adult	22 (35)	27 (42)	15 (23)
Paediatric	2 (15)	4 (31)	7 (54)
Modified Postural Drainage (no Head Down Tilt)			
Adult	46 (72)	11 (17)	7 (11)
Paediatric	7 (54)	5 (39)	1 (8)
Manual vibration			
Adult	46 (74)	12 (20)	4 (6)
Paediatric	11 (85)	2 (15)	0 (0)
Mechanical vibration			
Adult	9 (14)	42 (68)	12 (18)
Paediatric	3 (23)	8 (63)	2 (14)
Oscillating Positive Expiratory Pressure device			
Adult	62 (97)	2 (3)	0 (0)
Paediatric	12 (92)	1 (8)	0 (0)
Oscillating Positive Expiratory Pressure bubble/bottle			
Adult	53 (83)	10 (15)	1 (2)
Paediatric	11 (85)	1 (8)	1 (8)
Positive Expiratory Pressure mask or mouthpiece			
Adult	58 (90)	6 (10)	0 (0)
Paediatric	12 (92)	1 (8)	0 (0)
Percussion			
Adult	44 (69)	17 (26)	3 (5)
Paediatric	13 (100)	0 (0)	0 (0)
Physical exercise			
Adult	64 (100)	0 (0)	0 (0)
Paediatric	12 (92)	1 (8)	0 (0)
Positioning			
Adult	54 (86)	7 (11)	2 (3)
Paediatric	8 (67)	4 (33)	0 (0)
Sustained maximal inspiration			
Adult	21 (34)	30 (48)	11 (18)
Paediatric	NA	NA	NA

*NA* Not asked

In paediatrics, the techniques considered most effective were directed huffing (FET) (100%), PEP mask or mouthpiece (93%) and percussion (100%) (Table 4). The ACTs considered least effective were PD including head down tilt (54%) and mechanical vibration (14%).

### Factors influencing treatment decisions

The most commonly reported “extremely important factors” in choosing ACTs were ‘patient clinical presentation’ (72%) and ‘the presence/absence of contra-indications’ (72%) (Table 5). Factors most frequently considered “not at all important” were ‘taught at university’ (46%) and ‘medical staff’s preference’ (25%).

**Table 5:** Factors influencing choice of ACTs (n (%); *N* = 67)

Factors	Extremely important	Very Important	Moderately important	Slightly important	Not at all important
Patient clinical presentation e.g. sputum amount enquiries	48 (72)	16 (24)	3 (4)	0 (0)	0 (0)
Amount of oxygen/ventilatory support the patient is requiring	38 (57)	23 (34)	3 (4)	3 (4)	0 (0)
Presence/absence of precautions/contra-indications	48 (72)	15 (22)	3 (4)	1 (2)	0 (0)
Patient preference	33 (49)	29 (44)	5 (7)	0 (0)	0 (0)
Patients degree of dyspnoea or work of breathing	37 (55)	26 (39)	4 (6)	0 (0)	0 (0)
Personal experience/preference	14 (21)	30 (45)	17 (25)	4 (6)	2 (3)
Medical staff’s preference	0 (0)	8 (12)	19 (28)	23 (34)	17 (25)
Department/hospital policy	6 (10)	12 (18)	20 (30)	21 (32)	5 (8)
Highest level of evidence supporting a particular airway clearance technique	23 (34)	29 (43)	12 (18)	3 (5)	0 (0)
Influence from peers with experience in this area	7 (10)	30 (46)	19 (28)	7 (10)	4 (6)
Access to resources/equipment	23 (34)	32 (48)	12 (18)	0 (0)	0 (0)
Recent course attendance	5 (7)	14 (21)	21 (31)	17 (25)	9 (13)
Cost effectiveness	16 (24)	27 (40)	21 (31)	3 (5)	0 (0)
Time effectiveness	17 (25)	29 (43)	18 (27)	3 (5)	0 (0)
Taught at university	1 (2)	7 (10)	11 (17)	16 (25)	31 (46)

Most participants (71%) stated they were aware of the clinical guidelines [4] for bronchiectasis in children and adults in Australia and New Zealand. Regarding whether a change in practice at their place of work had occurred following guideline publication [4], 51% reported no change in practice, 31% were unsure if change occurred and 18% of participants reported a practice change. Reported changes in practice indicated via free text response included caution with the use of inhaled corticosteroids, greater use of autogenic drainage, more focus on hydration, increased confidence using prescribed exercise as part of ACT and increased medical acknowledgement of the role of physiotherapy and ACTs in management. Participants also reported that they were now less likely to trial nebulised hypertonic saline and felt that the guidelines reinforced the concept that tailoring ACTs to individual needs is appropriate.

Regarding knowledge of scientific evidence available for ACTs in this population, 23% of participants working with adults reported they were unsure what the current evidence indicated. In contrast, no participants working in the paediatric setting reported uncertainty regarding current evidence.

## Discussion

This survey is the first to investigate the current practice of physiotherapy with respect to use of ACTs for individuals experiencing an acute exacerbation of bronchiectasis. The most commonly used ACT varied depending on the age of the individual with bronchiectasis, however, directed huffing, physical exercise, the ACBT and PEP therapy were frequently used across most age groups, while modified postural drainage and percussion were most commonly applied in patients aged newborn-3 years. Participants perceived physical exercise, oscillating PEP therapy, directed huffing and the ACBT to be the ACTs most effective when working with adults, while directed huffing, PEP therapy and percussion were most effective when working with children. The important factors for choice of technique were patient presentation, and the presence/absence of contra-indications/precautions.

Most participants prescribed ACTs to their patients with an acute exacerbation of bronchiectasis, but the frequency and duration of intervention varied between adult and paediatric inpatients. This difference may be attributed to the greater ease with which adults can be taught an ACT and undertake this independently, while children require more supervision. It has been recommended that individuals admitted with an exacerbation of bronchiectasis are seen daily until their airway clearance is optimised, and that the duration is a minimum of 10 min and maximum of 30 min or until the patient becomes fatigued [6]. Participants adhered to guideline recommendations, with participants working with adults reporting the provision of one or two sessions per day, most commonly of 11–15 min duration, while most paediatric physiotherapists treated individuals twice daily, most commonly for 16–20 min. This is similar to a previous survey, which reported most child participants with bronchiectasis completed airway clearance therapy at least once per day when unwell [14].

The most commonly implemented ACTs in an adult population were the ACBT, directed huffing and physical exercise, similar to previous surveys in bronchiectasis [11, 15]. Additionally, all three techniques were perceived effective by more than 90% of participants. However, there is little evidence available for the use of the ACBT or physical exercise in individuals with exacerbations of bronchiectasis [13]. The ACBT and huffing are commonly used ACTs clinically as they require no equipment, can be completed in a range of positions and can be adapted to the patient and their presenting condition [20]. The ACBT has been investigated in one study with individuals with an exacerbation of bronchiectasis, and was found to be superior in sputum expectoration (greater decrease in 24 h collection), cough-related quality of life and gas exchange when compared to more traditional ACTs of percussion and postural drainage [21]. Physical exercise as an ACT has not been investigated in individuals with an exacerbation of bronchiectasis, but is a growing area of research in cystic fibrosis [22, 23]. With a high frequency of use of this ACT in adults and paediatrics, this may be an important area for future research.

Participants in this study also reported greater use of PEP and oscillating PEP devices when compared to a previous study from the United Kingdom in 2002 [11]. In the previous study [11] PEP was used routinely by 7% of participants and oscillating PEP by 12% of participants, in the current study 41% of participants were using PEP therapy routinely and 75% of participants were using an oscillating PEP device routinely. This difference may be due to an increase in availability of these devices, patient preference to complete ACTs independently, and an increase in evidence for the safety and efficacy of these devices in individuals with stable bronchiectasis [12, 24]. Interestingly, oscillating PEP was perceived very effective or effective by 97% of participants in this study, however there is little evidence available for the use of oscillating PEP for individuals with an exacerbation of bronchiectasis. There have been two small studies [25, 26] which found that oscillating PEP therapy was preferred by participants over other types of ACTs. While this may be associated with increased sputum production during a treatment session, this result was not significant in either study [25, 26]. A recent international guideline also recommended implementation of the ACBT or oscillating PEP for individuals with bronchiectasis [6]. As individuals with bronchiectasis appear to prefer oscillating PEP therapy, and most participants in this study perceived this to be an effective ACT, future research could explore the effect of oscillating PEP therapy, compared to the ACBT, for individuals with an exacerbation of bronchiectasis.

Another change in ACT prescription is the use of postural drainage. A previous survey study from 2002 [11] demonstrated that most participants used postural drainage routinely, while participants in this survey reported rarely using postural drainage incorporating a head down tilt in the adult population. This shift may reflect the finding of increased episodes of reflux in cystic fibrosis, with other treatment techniques taking preference [27]. Another potential explanation is that the recommendations for ACTs now promote active involvement of the patient, rather than passive techniques [28].

The ACTs listed in the survey were based on previous surveys from the United Kingdom and Australia and New Zealand [11, 14, 15] and show a preference to ACTs which are more frequently used in this part of the world. Although participants were given the option of an open, ‘other’, question to capture information from participants who used ACTs that were not listed, no participants in this study listed other ACTs used more frequently in other parts of the world, such as expiration with an open glottis in the lateral posture (ETGOL). This ACT has been shown to be effective to facilitate secretion removal, reduce exacerbations and improve quality of life for individuals with bronchiectasis, and so should be included in future surveys investigating current practice [29].

The ACTs used by participants serving a paediatric population varied according to age group. Adherence to ACTs can be a major problem for children and adolescents with bronchiectasis, and so ensuring techniques are age appropriate is important [17, 30]. In the newborn-3 years age range, techniques used were passive techniques, appropriate for this age [17]. Regarding management of individuals aged 4–17 years, participants reported the common use of directed huffing, physical exercise, PEP therapy and the ACBT. This contrasts with a previous survey of New Zealand children and their families with bronchiectasis [14], with families reporting using PD (modified or including head down tilt), ACBT or PEP therapy. The difference in ACT choice between studies may be due to local practice, therapist’s preference, or the availability of devices. The previous study was based in one hospital district, while the current study included participants from multiple locations across Australia and New Zealand; this may account for variation in clinical practice. Additionally, the reports of physiotherapists managing individuals with bronchiectasis may vary from the reports of children and their families living with bronchiectasis.

The most important factors influencing ACT choice include clinical presentation and presence of contra-indications. This mirrors what has been reported by physiotherapists managing acute exacerbations of COPD [16]. The least important factors for choice of ACT were similar to a previous report, being medical staff preference and training [11]. This implies that physiotherapists are using assessment and clinical reasoning skills when deciding which ACT to prescribe. The importance of implementing techniques that are evidence based has been highlighted in the clinical guidelines for bronchiectasis (6,7), yet physiotherapists who completed this survey are routinely using, and perceive effective ACTs underpinned by minimal evidence. In the absence of high-quality research, physiotherapists must rely on other factors when deciding which ACT to use for a particular patient. It appears, that participants’ choice of ACT is impacted by access to resources, perceived time and cost effectiveness of an ACT and personal experience.

There are limitations to this study. The first is the low or unknown response rate due to the method of survey distribution. The number of physiotherapists who received the invitation to participate and met the eligibility criteria is unknown. However, the final number of responses received (121) was very similar to the raw numbers achieved in previous surveys of Australian and New Zealand physiotherapists working with individuals with chronic respiratory conditions [15, 16]. Furthermore, a previous study investigating ACTs services for individuals in Australia reported 184 services available, which is similar to the final number of responses received, however it is unable to be determined how many different services are represented in this study [31]. The survey remained open for 8 months to maximise participation numbers, however, this may have influenced the data collected, as clinical practice may have changed over time. Approximately 40% of participants had less than 5 years of respiratory experience. Clinical experience and exposure are likely to influence practice, and this may account for the selection of ACTs, perceptions of efficacy, and factors influencing treatment decisions. The response numbers to the paediatric section were lower than for the adult population, which may be due to a smaller proportion of physiotherapists working with this population, or, the survey length, with incomplete responses to questions increasing as participants progressed through the survey. Although there were participants across Australia and New Zealand, and a similar number of responses compared to previous studies completed in respiratory conditions, it is unknown if the data accurately represents current practice throughout these countries. Additionally, the data is self-reported and predominantly based on multiple choice questions and Likert scales of perceived use and effectiveness. It is well documented that clinical reasoning and decision-making regarding treatment interventions are complex processes [32, 33], so this is difficult to measure in survey form, therefore some complexities of participants’ actual clinical practice may not have been identified. No timeframe for participants to consider when answering each question was included, and this should be considered in future surveys. Data regarding inpatient versus outpatient management are unable to be compared. It is reasonable to believe that practices may differ between inpatient and outpatient settings and this should be considered in future surveys.

## Conclusions

Most participants working with adults and all participants working in paediatrics reported they prescribed ACTs 81–100% of the time when managing an individual with an acute exacerbation of bronchiectasis. The most commonly used ACTs with adults were directed huffing, physical exercise, the ACBT, positioning and oscillating PEP devices. Within paediatric populations, the most used ACT varied depending on age group. All participants believed that directed huffing, physical exercise, oscillating PEP devices, the ACBT and PEP therapy were effective. Patient clinical presentation was the most commonly reported important factor for determining choice of ACT. The findings from this survey will assist in guiding future research regarding the clinical efficacy of ACTs for adults and paediatrics with an acute exacerbation of bronchiectasis.

## Data Availability

The datasets used and/or analysed during the current study are available from the corresponding author on reasonable request.

## Abbreviations

ACBT: Active cycle of breathing technique
ACT: Airway clearance technique
ETGOL: Expiration with the glottis open in lateral posture
FET: Forced expiratory technique
HDT: Head down tilt
HRQOL: Health related quality of life
NA: Not asked
PD: Postural drainage
PEP: Positive expiratory pressure

## References

[1] Barker AF (2002). Bronchiectasis. N Engl J Med.

[2] O'Neill K, O'Donnell AE, Bradley JM (2019). Airway clearance, mucoactive therapies and pulmonary rehabilitation in bronchiectasis. Respirology.

[3] Chalmers JD, Aliberti S, Blasi F (2015). Management of bronchiectasis in adults. Eur Respir J.

[4] Chang AB, Bell SC, Torzillo PJ, Byrnes CA, Maguire GP, Holland AE (2015). Thoracic Society of Australia and New Zealand guidelines: chronic suppurative lung disease and bronchiectasis in children and adults in Australia and New Zealand. Med J Australia.

[5] Pasteur MC, Bilton D, Hill AT, Group BTSBnCFG (2010). British Thoracic Society guideline for non-CF bronchiectasis. Thorax.

[6] Hill AT, Sullivan AL, Chalmers JD, De Soyza A, Stuart Elborn J, Andres Floto R (2019). British Thoracic Society guideline for bronchiectasis in adults. Thorax.

[7] Polverino E, Goeminne PC, McDonnell MJ, Aliberti S, Marshall SE, Loebinger MR, et al. European Respiratory Society guidelines for the management of adult bronchiectasis. Eur Respir J. 2017;50(3). 10.1183/13993003.00629-2017. 10.1183/13993003.00629-2017 28889110

[8] Pereira MC, Athanazio RA, Dalcin PTR, Figueiredo MRF, Gomes M, Freitas CG (2019). Brazilian consensus on non-cystic fibrosis bronchiectasis. J Bras Pneumol.

[9] Bott J, Blumenthal S, Buxton M, Ellum S, Falconer C, Garrod R (2009). Guidelines for the physiotherapy management of the adult, medical, spontaneously breathing patient. Thorax.

[10] Snijders D, Fernandez Dominguez B, Calgaro S, Bertozzi I, Escribano Montaner A, Perilongo G (2015). Mucociliary clearance techniques for treating non-cystic fibrosis bronchiectasis: is there evidence?. Int J Immunopathol Pharmacol.

[11] O'Neill B, Bradley JM, McArdle N, MacMahon J (2002). The current physiotherapy management of patients with bronchiectasis: a UK survey. Int J Clin Pract.

[12] Lee AL, Burge AT, Holland AE (2015). Airway clearance techniques for bronchiectasis. Cochrane Database Syst Rev.

[13] Phillips J, Lee A, Pope R, Hing W. Effect of airway clearance techniques in patients experiencing an acute exacerbation of bronchiectasis: a systematic review. Physiother Theor Pract. 2019:1–16. 10.1080/09593985.2019.1579286. 10.1080/09593985.2019.1579286 30776932

[14] Butler SG, Hill LJ, Harrison J, Reed P, Nikora G, Takai C (2008). Is there a difference in airway clearance practices between children with non cystic fibrosis bronchiectasis and cystic fibrosis?. NZ J Physiother.

[15] Lee A, Button B, Denehy L (2008). Current Australian and New Zealand physiotherapy practice in the management of patients with bronchiectasis and chronic obstructive pulmonary disease. NZ J Physiother.

[16] Osadnik CR, McDonald CF, Holland AE (2013). Airway clearance techniques in acute exacerbations of COPD: a survey of Australian physiotherapy practice. Physiotherapy.

[17] Lee AL, Button BM, Tannenbaum EL (2017). Airway-clearance techniques in children and adolescents with chronic suppurative lung disease and bronchiectasis. Front Pediatr.

[18] Australian Physiotherapy Association. 2016 annual report: Australian Physiotherapy Association; 2017. https://australian.physio/aboutus/annual-reports. Accessed 27 May 2020.

[19] Physiotherapy New Zealand (2018). Annual report 2017.

[20] Lewis LK, Williams MT, Olds TS (2012). The active cycle of breathing technique: a systematic review and meta-analysis. Respir Med.

[21] AbdelHalim HA, AboElNaga HH, Fathy KA (2016). Comparison between active cycles of breathing with postural drainage versus conventional chest physiotherapy in subjects with bronchiectasis. Egypt J Chest Dis Tuberc.

[22] Ward N, Stiller K, Holland AE, Bingham J, Bishop J, Button B (2019). Exercise is commonly used as a substitute for traditional airway clearance techniques by adults with cystic fibrosis in Australia: a survey. J Physiother.

[23] Ward N, Stiller K, Holland AE (2019). Exercise as a therapeutic intervention for people with cystic fibrosis. Expert Rev Respir Med.

[24] Lee AL, Burge AT, Holland AE (2017). Positive expiratory pressure therapy versus other airway clearance techniques for bronchiectasis. Cochrane Database Syst Rev.

[25] Patterson JE, Hewitt O, Kent L, Bradbury I, Elborn JS, Bradley JM (2007). Acapella versus ‘usual airway clearance’ during acute exacerbation in bronchiectasis: a randomized crossover trial. Chron Respir Dis.

[26] Tsang S, Jones A (2003). Postural drainage or flutter device in conjunction with breathing and coughing compared to breathing and coughing alone in improving secretion removal and lung function in patients with acute exacerbation of bronchiectasis: a pilot study. Hong Kong Physiother J.

[27] Button BM, Heine RG, Catto-Smith AG, Phelan PD, Olinsky A (1997). Postural drainage and gastro-oesophageal reflux in infants with cystic fibrosis. Arch Dis Child.

[28] Rand S, Prasad SA (2012). Exercise as part of a cystic fibrosis therapeutic routine. Expert Rev Respir Med.

[29] Muñoz G, Gracia J, Buxó M, Alvarez A, Vendrell M. Long-term benefits of airway clearance in bronchiectasis: a randomised placebo-controlled trial. Eur Respir J. 2018;2018(51). 10.1183/13993003.01926-2017. 10.1183/13993003.01926-2017 29326318

[30] Chang AB, Bush A, Grimwood K (2018). Bronchiectasis in children: diagnosis and treatment. Lancet.

[31] Cooper L, Johnston K, Williams M (2019). Airway clearance services (ACSs) in Australia for adults with chronic lung conditions: scoping review of publicly available web-based information. BMC Health Serv Res.

[32] Smith M, Higgs J, Ellis E (2008). Characteristics and processes of physiotherapy clinical decision making: a study of acute care cardiorespiratory physiotherapy. Physiother Res Int.

[33] Holdar U, Wallin L, Heiwe S (2013). Why do we do as we do? Factors influencing clinical reasoning and decision-making among physiotherapists in an acute setting. Physiother Res Int.

